# Lung Radiofrequency Ablation: Potential as a Therapy to Oligometastasis and Oligorecurrence

**DOI:** 10.1155/2012/196173

**Published:** 2012-10-18

**Authors:** Takao Hiraki, Susumu Kanazawa

**Affiliations:** Department of Radiology, Okayama University Medical School, 2-5-1 Shikatocho, Kitaku, Okayama 700-8558, Japan

## Abstract

The early results (e.g., patient survival) of RFA for the treatment of patients with NSCLC and pulmonary metastasis from various primary lesions including colorectal cancer, lung cancer, hepatocellular carcinoma, renal cell carcinoma, and sarcoma appear encouraging and suggest the potential to offer long-term survival for the patients with oligorecurrence or oligometastasis of lung cancer. The usefulness of RFA for oligorecurrence or oligometastasis of lung cancer should be clarified by prospective studies in the future.

## 1. Introduction

Primary lung cancer is the most common malignancy and the leading cause of death from cancer worldwide. In addition, the lungs are the second most frequent site of metastasis from extrathoracic cancers and the only site of metastasis in 20% of such cases. Surgical resection is the first-line treatment for nonsmall-cell lung cancers (NSCLC) and offers the best treatment opportunity. Surgery is also accepted as a treatment option for carefully selected patients with metastatic lung cancer. However, surgical resection is not suitable for many patients mainly because of the advanced stage of cancer, compromised lung function, and/or comorbidities. Although chemotherapy, radiation therapy, or a combination of these serves as alternative treatments for such patients, complete remission of the disease is rarely achieved. Therefore, research that focused on alternative therapies for lung cancer has been extensive in the past decades; such therapies include stereotactic radiation therapy, cryoablation, laser ablation, and radiofrequency (RFA).

RFA causes focal coagulation necrosis of tissue by delivery of energy in the form of an alternating electrical current with a frequency of 460 to 500 kHz in the range of radio waves. The location of the ablative effect is determined by the precise placement of the radiofrequency electrode, usually using imaging guidance. The radiofrequency electrical current is concentrated near the noninsulated tip of the electrode, and the circuit is completed by returning either to electrical grounding pads usually located on the patient's thighs. The alternating electrical current causes ionic dipolar molecules in surrounding tissue and fluids to agitate, resulting in frictional heating that is greatest adjacent to the noninsulated portion of the electrode. The heat energy is then distributed radially to surrounding tissues. When radiofrequency current is applied in a slow, controlled fashion, the tissue heating is local, typically ellipsoid in shape, and predictable in distribution. 

At first, RFA was noted as a therapy for hepatocellular carcinoma. The favorable outcomes of the RFA in the liver have encouraged the application of this technique to cancer in other organs. In 2000, Dupuy et al. [1] firstly reported clinical application of this technique in the lung. Since then, RFA has been gaining popularity rapidly as a treatment of lung cancer. RFA of lung cancer is usually performed under CT-guidance and the techniques are quite simple and similar to those used for CT-guided lung biopsy. Herein, we review clinical outcomes of RFA of lung cancer and discuss the potential to be used as a therapy to oligometastasis and oligorecurrence.

## 2. Rationale for RFA of Oligometastasis **** and Oligorecurrence

Oligometastasis and oligorecurrence, proposed by Niibeand Hayakawa [2], are the condition of one or a few metastatic or recurrent lesions without and with controlled primary tumor, respectively. Although significance of local therapy of metastatic lesions for survival benefit may be controversial, the International Registry of Lung Metastases (IRLM) [3] reported that 5-year overall survival for patients with complete resection of metastatic lung tumors was 36%, compared with 13% for patients without it. Further, for the patient for whom lung metastases were completely resected, survival depended on tumor number; that is, smaller number of metastases indicated better survival. Such data may suggest the rationale for applying local therapy including RFA for oligometastasis and oligorecurrence. The registry also reported that the patients with disease-free intervals of 36 months or more had better prognosis. Thus, the patients with slow growing tumors are more appropriate candidates for RFA.

## 3. RFA of Primary Lung Cancer

There have been several studies on RFA in the management of primary lung cancers. In 2007, Simon et al. [4] reviewed 75 cases of previously untreated stage I NSCLC, resulting in overall survival of 78%, 57%, and 27% at 1, 2, and 5 years, respectively. Those results seemed to compare favorably with previous studies using external beam radiotherapy in similar stage tumors. Survival was significantly associated with tumor size, with approximately 50% of 5-year survival for the patients with tumors <3 cm. Further encouraging results were reported in a prospective multicenter study by Lencioni and coworkers [5]. Their study included 33 patients with NSCLC treated with RFA; of those, 13 patients had medically inoperable stage I NSCLC. The overall survival in patients with NSCLC was 70% and 48% at 1 and 2 years, respectively, with cancer-specific survival of 92% and 73% at 1 and 2 years. Subgroup analysis revealed 2-year overall survival of 75% and 2-year cancer-specific survival of 92% in patients with inoperable stage I NSCLC. Hiraki et al. [6] reported the outcomes of 27 patients with stage I NSCLC who were treated with RFA. During median follow-up period of 22 months, the mean survival time was 42 months. The overall survival and cancer-specific survival rates were 90% and 100% at 1 year, 84% and 93% at 2 years, and 74% and 83% at 3 years, respectively. Most recently, Hiraki et al. [7] have updated their data using 50 patients with stage I NSCLC. During median follow-up period of 37 months, a median survival time was 67 months, the overall, cancer-specific and disease-free survivals were 94%, 100%, and 82% at 1 year, 86%, 93%, and 64% at 2 years, and 74%, 80%, and 53% at 3 years, respectively. Despite favorable survival data, local progression was observed in 16 (31%) of the 52 tumors. Lanuti et al. [8] reported that during a median follow-up of 17 months, median survival time was 30 months for 31 patients; survaial rate was 85% at 1 year, 78% at 2 years, and 47% at 3 years; local progression rate was 32%. Pennathur et al. [9] reported that during a mean follow-up of 29 months, survival rate for 19 patients was 95% at 1 year, and 68% at 2 years; local progression rate was 42%. 

With regard to oligorecurrence of NSCLC, Kodama et al. [10] carried out an interesting study. Their study included 44 patients who underwent lung RFA for recurrent NSCLC after surgery. Forty-three patients had no extrapulmonary metastasis; one patient had liver and splenic metastasis, which was also treated with RFA. Single or multiple intrapulmonary recurrences were ablated. During mean follow-up period of 29 months, the overall survival rates were 98% at 1 year, 73% at 2 years, and 56% at 3 years. The recurrence-free survival rates were 77% at 1 year and 41% at 3 years. Tumor size and sex were independent significant predictors in the multivariate analysis. This study indicated that RFA may offer a chance of long-term survival for the patients with oligorecurrence of primary lung cancer.

## 4. RFA of Metastatic Lung Cancer

### 4.1. Metastasis from Colorectal Cancer

 The cancer that most frequently metastasizes to the lung is colorectal cancer. Approximately 10% of the patients who undergo curative resection for colorectal cancer develop lung metastases [11]. Standard treatment options include surgical resection and chemotherapy. Many surgeons believe that surgical resection is the best treatment that offers the potential for long-term survival in selected patients. Several large studies on pulmonary metastasectomy have demonstrated similar survival after surgery, with approximately 40% of the 5-year survival rate. Further, systematic review of 1684 patients by Pfannschmidt et al. [12] showed 48% of 5-year survival. However, patients with pulmonary metastases are often nonsurgical candidates because of other coexistent metastases, poor cardiopulmonary function, or refusal to undergo surgery. A recent chemotherapy regimen using fluorouracil and leucovorin with irinotecan or oxaliplatin has been shown to prolong survival, but the long-term results are still less than satisfactory, with a median survival of 14.8–21.5 months for the patients with metastatic colorectal cancer [13]. 

The prospective multicenter study by Lencioni et al. [5] showed that overall survival rate was 89% at 1 year and 66% at 2 years in patients with colorectal metastases; cancer-specific survival was 91% at 1 year and 68% at 2 years. Hiraki et al. [14] also assessed survival rates for 27 patients with pulmonary metastases from colorectal cancer. During the median follow-up period of 20.1 months after RFA, the overall survival rates were 96% at 1 year, 54% at 2 years, and 48% at 3 years. The most significant prognostic factor was the presence of extrapulmonary metastasis at the time of RFA. While patients with extrapulmonary metastasis never survived for 2 years, survival rates for patients without extrapulmonary metastasis were favorable, indicating 100% at 1 year, 76% at 2 years, and 68% at 3 years. These results showed the potential of long-term survival of the patients with oligorecurrence from colorectal cancer with RFA. Yamakado et al. [15] reported the outcomes of a retrospective multicenter study on RFA for pulmonary metastases from colorectal cancer. The estimated 3-year survival rate was 46% for all patients. Extrapulmonary metastasis, tumor size, and the carcinoembryonic antigen level were significant prognostic factors in the univariate analysis. The first two factors were significantly independent prognostic factors in the multivariate analysis. Thirty-six patients with small lung metastases (< or =3 cm) and no extrapulmonary metastases had a 3-year survival rate of 78%. Yamakado et al. [16] also reported single center experiences of RFA for pulmonary metastases from colorectal cancer. For 78 patients, the 1-, 3-, and 5-year survival rates were 84%, 56%, and 35%, respectively, during a mean follow-up period of 25 months. The median survival time was 38.0 months. Univariate analysis revealed maximum tumor diameter of 3 cm or less, single-lung metastasis, lack of extrapulmonary metastasis, and normal carcinoembryonic antigen (CEA) level as better prognostic factors. The latter two were significant independent prognostic factors. The 1-, 3-, and 5-year survival rates were 97.7% (95% CI, 93.3–100%), 82.5% (95% CI, 68.2–96.8%), and 57.0% (95% CI, 34.7–79.2%) in 54 patients with no extrapulmonary metastases and 96.9% (95% CI, 90.8–100%), 86.1% (95% CI, 71.1–100%), and 62.5% (95% CI, 36.3–88.6%) in 33 patients with negative CEA levels. More recently, Chua et al. [17] reported promising long-term outcome obtained by a prospective trial of 108 patients with pulmonary metastases from colorectal cancer. The median survival reached 60 months, which appeared equivalent to data obtained by metastasectomy. 

### 4.2. Metastasis from Hepatocellular Carcinoma

Hiraki et al. [18] performed a retrospective multicenter study on RFA for pulmonary metastases from hepatocellular carcinoma HCC. This study included 32 patients who had no intrahepatic recurrence or had treatable intrahepatic recurrence, who had no other metastases, and for whom RFA was performed with curative intent (i.e., not palliatively). The overall survival rates were 87% at 1 year and 57% at 2 and 3 years during a median follow-up period of 20.5 months. Median and mean survival times were 37.7 months and 43.2 months, respectively. Significantly better survival rates were obtained for patients with an absence of viable intrahepatic recurrence, Child-Pugh grade A, absence of liver cirrhosis, absence of hepatic C virus infection, and *α*-fetoprotein level of 10 ng/mL or lower at the time of RFA. These results seem to suggest that pulmonary metastasis from HCC is suitable candidates for RFA, if primary cancer is well controlled (i.e., oligorecurrence). 

### 4.3. Metastasis from Renal Cell Carcinoma

In cases of pulmonary metastases from renal cell carcinoma, patient survival was evaluated using data from 2 institutions [19]. This study included 39 nonsurgical candidates who were divided into 2 groups: a curative ablation group, which was formed by 15 patients with 6 or fewer lung metastases measuring ≤6 cm that were confined to the lung and who had all lung tumors ablated, and the palliative ablation group, which included 24 patients with extrapulmonary lesions, 7 or more lung tumors, or large tumors of >6 cm, and who had mass reduction. The overall survival rates in the curative and palliative ablation groups were 100% and 90% at 1 year, 100% and 52% at 3 years, and 100% and 52% at 5 years, respectively. The maximum lung tumor diameter was a significant prognostic factor.

### 4.4. Metastasis from Sarcoma

Palussière et al. [20] reported the outcomes of RFA for pulmonary metastases from various kinds of sarcoma. This study included 29 patients with a maximum of 5 lung metastases and without extrapulmonary metastasis (i.e., oligorecurrence). During median follow-up period of 50 months, the 1- and 3-year survival rates were 92.2% and 65.2%, respectively. Median disease-free survival was 7 months. This study suggests that RFA may offer a chance for long-term survival for patients with oligorecurrence from sarcoma, although the disease may recur in a relatively short-term followup. 

Nakamura et al. [21] reported on RFA for 20 patients with pulmonary metastases from musculoskeletal sarcomas. During the mean follow-up period of 18 months (range, 7 months to 54 months), 9 of 20 patients died of lung tumor progression. The 1- and 3-year survival rates from RF ablation were 58% and 29% with a median survival time of 12.9 months in all patients. Survival rate for 14 patients with controlled primary tumor (33% at year) was not significantly different from that for 6 patients without controlled primary tumor (52% at 1 year). Survival rate for 10 patients with ≤5 lung metastases (38% at year) was not significantly different from that for 10 patients with >5 lung metastases (88% at 1 year). Thus, survival did not seem to depend on whether oligorecurrence or not in the population that they studied. 

## 5. Advantages and Limitations of RFA

Major limitation of RFA may be limited local efficacy. RFA induces various complications. Food and Drug Administration in the United States made a public announcement regarding deaths following RFA of lung tumors in 2007. Rare but serious complications may occur including bronchopleural fistula [22], pulmonary artery pseudoaneurysm [23], systemic air embolism [24], injury of the brachial nerve and the phrenic nerve [25, 26], pneumonia [27], and needle-tract seeding of cancer [28]. A case of fatal acute deterioration of interstitial pneumonia after RFA has been also reported [29]. Survey is required to recognize an incidence of acute deterioration after RFA in the patients with interstitial pneumonia and thereby to determine a role of RFA in such patients. 

Notable advantages of RFA include limited influence on pulmonary function. According to a report by Ambrogi et al. [30], the mean forced vital capacity (VC) was 2.63 and 2.80 L at 1 and 3 months, respectively, compared with 2.91 L before RFA; the mean forced expiratory volume in 1s (FEV(1)) was 1.71 and 1.86 L at 1 and 3 months, respectively, compared with 1.97 L before RFA. The multicenter prospective study by Lencioni et al. [5] also showed mean forced VC and FEV1 of 2.6 and 1.7 L, respectively, at 1 month, compared with 2.9 and 1.9 L, respectively, before RFA in 22 patients with non-small cell lung cancer. Tada et al. [31] reported that the mean VC and FEV(1) before RFA and 1 and 3months after RFA were 3.04 and 2.24L, 2.79 and 2.11L, and 2.85 and 2.13L, respectively. De Baère et al. [32] reported that pulmonary function did not decrease after RFA; the mean VC and FEV1 were 2.9 and 2.2 L, respectively, after RFA, compared with 2.9 and 2.2 L, respectively, before RFA. 

The freedom to perform the procedure regardless of any previous therapy is another important advantage. Adhesion after pulmonary surgery or radiation-induced pneumonitis is not an obstacle for performing the procedure. Thus, the procedure may be used as a salvage treatment for oligorecurrence after surgery and radiation therapy. At the same time, RFA procedure is not an obstacle for performing concurrent or adjuvant chemotherapy or adjuvant radiation therapy. According to the Norton-Simon hypothesis [33], the effectiveness of chemotherapy agents is proportional to the growth rate of the tumor and the fastest tumor growth rates occur when tumors are not bulky. Therefore, if RFA can downsize the primary tumor, the remaining tumor cells may become more sensitive to chemotherapy. The combination with such therapeutic modalities is expected to increase the efficacy of RFA not only through an additive effect but also due to synergistic effects [34]. The availability to repeat procedures whenever required is also an important advantage. Although RFA results in relatively high rate of local failure, local failure may be salvaged by repetition of the procedure [35]. 

## 6. Conclusions

In conclusion, the early results of RFA for the treatment of patients with NSCLC and pulmonary metastasis from various primary cancers appear encouraging and suggest the potential to offer long-term survival for the patients with oligorecurrence or oligometastasis of lung cancer. The usefulness of RFA for oligorecurrence or oligometastasis of lung cancer should be clarified by prospective studies in the future.

## Abbreviation

RFA: Radiofrequency ablation.
